# Advancements in mapping, cardiac imaging, and ablation techniques for the management of ventricular tachycardias

**DOI:** 10.1007/s10840-026-02336-4

**Published:** 2026-04-22

**Authors:** Alejandro Sanchez-Nadales, Richard Amoateng, Aamir Husain Twing, Andres Sanchez-Nadales, Khaled Abdelhady, Sukit Ringwala, Dana Johnson

**Affiliations:** 1 Department of Medicine-Clinical Cardiac Electrophysiology, University of Illinois Chicago, Chicago, IL 60607 USA; 2 Department of Medicine-Cardiovascular Diseases, University of Illinois Chicago, Chicago, IL 60607 USA; 3 Department of Cardiothoracic Surgery, University of Illinois Chicago, Chicago, IL 60612 USA

**Keywords:** Cardiac magnetic resonance, Electro-anatomical mapping, Intracardiac echocardiography, Cardiac computed tomography, Pulse field ablation, Surgical ablation, Ventricular tachycardia

## Abstract

Ventricular tachycardias represent a complex clinical challenge, particularly due to the intricate arrhythmic circuits and diverse substrates found in ischemic and non-ischemic cardiomyopathies. This review explores the significant advancements in mapping, imaging, and ablation technologies that have transformed the management strategies for ventricular arrhythmias. Recent innovations such as pulsed-field ablation and high-resolution imaging modalities like cardiac magnetic resonance and multi-detector computed tomography have refined our approach to ablate ventricular tachycardias. The evolution of surgical and hybrid ablation techniques also highlights an adaptive response to cases unmanageable by conventional interventions. Furthermore, we discuss the integration of new diagnostic tools and detailed substrate analysis, which are pivotal in enhancing procedural outcomes and reducing recurrence. This synthesis of current practices with emerging technologies offers a promising direction toward more effective and personalized management of VT, underlining the need for continuous advancement in both technological capabilities and procedural strategies.

## Introduction

Ventricular tachycardias (VTs) present significant management challenges, with traditional therapies like antiarrhythmic drugs (AAD) and implantable cardioverter-defibrillators (ICD) often failing to modify the arrhythmic substrate fundamentally. Consequently, catheter ablation has emerged as a pivotal modality for altering the arrhythmic circuitry directly. The 2019 expert consensus advocates for catheter ablation when AAD proves ineffective or is poorly tolerated or if the patient prefers this option [1]. It is particularly recommended as first-line therapy for symptomatic ventricular premature depolarizations, VT arising from the right ventricular outflow tract in structurally normal hearts, and bundle branch reentrant VT. Strong evidence supports ablation in patients with ischemic heart disease and ICD to mitigate the recurrence of monomorphic VT episodes (Class IIb, Level of Evidence: A) [1], thereby reducing associated risks and avoiding the long-term side effects of AAD and the adverse effects of multiple ICD shocks in complex cases of structural heart disease.

Recent advancements in ablation technologies and cardiac imaging have significantly enhanced the precision and safety of VT ablations [2]. Nonetheless, the intricate anatomy of ventricular substrates, often involving transmural and intramural pathways, continues to challenge the efficacy of conventional radiofrequency ablation techniques [3]. This complexity is evidenced by significant rates of VT recurrence and procedural complications [4], highlighting the need for more effective and sustainable ablation strategies. The advent of high-resolution cardiac imaging techniques such as cardiac magnetic resonance (CMR) and multidetector computed tomography (MDCT) has revolutionized pre-procedural planning by providing detailed visualizations of myocardial scars critical for guiding ablation strategies [5]. Additionally, the development of novel ablation approaches like hybrid surgical and pulse field ablation (PFA) and advanced mapping promises to overcome some limitations of traditional methods [6]. As the field stands on the cusp of these technological evolutions, reviewing current practices, assessing emerging technologies, and identifying future directions for managing VT is imperative.

### The role of different mapping techniques

**Fig. 1:**
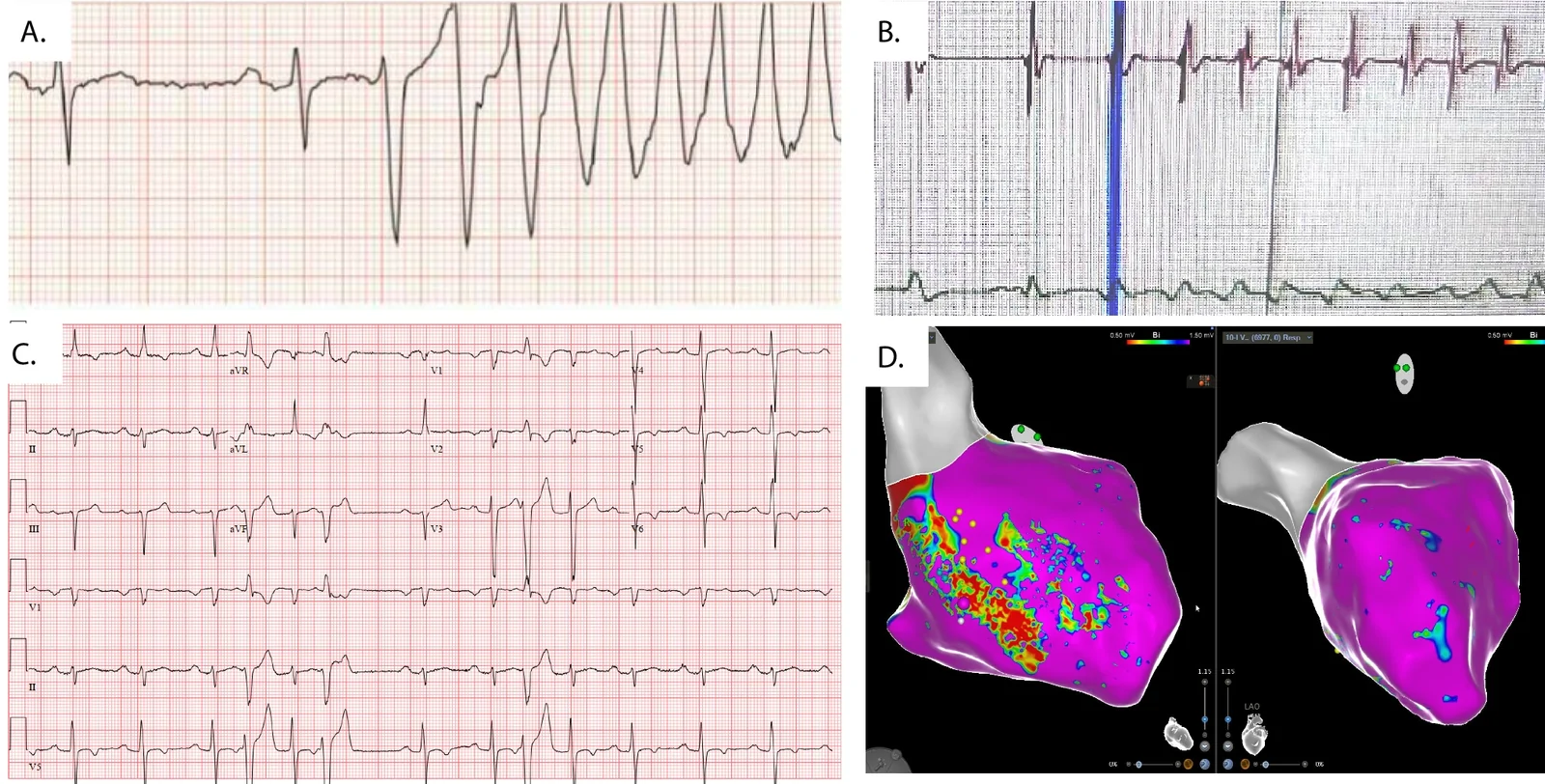
Multimodality approach to ablation: **A**. Inpatient telemetry showing low burden PVC initiate VT. **B**. ICD tracing correlates to PVC in **C**. which was mapped to distal fascicle, marked by pink tag in **D**. Ablation here led to non-inducibility

Effective planning prior to a procedure is crucial for anticipating challenges and determining optimal mapping and ablation strategies. An essential tool in this preparatory phase is the 12-lead electrocardiogram (ECG), which aids in pinpointing the likely origin of VT [5]. The insights gained from the ECG guide the focus of mapping efforts. For patients equipped with ICDs, analyzing the timing and specifics of VT episodes recorded by the device provides additional clues, differentiating VT from other cardiac rhythms that may occur during the procedure, especially when a detailed 12-lead ECG is unavailable (Fig. 1).

The primary objective of VT ablation is to target the specific pathways used by VT within the heart or the initial points of these arrhythmias in cases of focal VT, such as ventricular premature depolarizations (VPDs). This targeting is accomplished through four principal mapping techniques: activation, entrainment, pace, and substrate mapping. Each method is selected based on the particular characteristics of the VT, ensuring the ablation procedure is as effective as possible [6].

#### Activation mapping

Activation mapping plays a crucial role in analyzing the sequence of myocardial activation during stable VT or VPDs. This technique involves recording local electrograms at various ventricular sites and comparing their activation times to a reference, usually the onset of the QRS complex during the arrhythmia [7]. Electroanatomic mapping systems (EAM) are utilized to visually represent these timings in color, effectively highlighting the earliest sites of activation. Such visualizations are particularly valuable for pinpointing the origins of focal arrhythmias. Pre-QRS electrograms, typically occurring 30–50 ms prior to the QRS, are critical for identifying the arrhythmia's origin. The detection of a unipolar QS pattern in the local electrogram confirms these findings, indicating outward activation from the catheter tip [7].

In scenarios involving scar-related re-entry VT, activation mapping is helpful in identifying critical isthmuses. These are characterized by diastolic activation with low-amplitude, fractionated electrograms, indicative of slow conduction through scarred tissue [8]. However, despite the effectiveness of activation mapping, its comprehensive application is often hindered by the hemodynamic instability of VT. This limitation makes thorough mapping feasible in only 10% to 30% of VT cases [9].

#### Entrainment mapping

Entrainment mapping is crucial for analyzing re-entrant VT circuits, especially when VT is hemodynamically stable. This technique involves pacing at specific sites that display mid-diastolic or pre-systolic activities, thereby confirming their involvement in the re-entrant circuit [10]. Originating over three decades ago, entrainment mapping is based on the understanding that VT circuits can involve complex structures, such as Figure-of-8 re-entry, which contain both critical and noncritical components. The critical components (namely the entrance, isthmus, and exit) are vital for sustaining the arrhythmia and are typically anatomically distinct, making them prime targets for ablation.

During entrainment, pacing that effectively captures the circuit will replicate the spontaneous VT's QRS configuration, a phenomenon known as concealed fusion. This indicates effective engagement with the circuit. Key sites are identified by a post-pacing interval (PPI) that closely matches the tachycardia cycle length (TCL), along with a corresponding local activation sequence. These critical sites are categorized based on their stimulus-to-QRS (S-QRS) interval relative to the TCL: exit sites exhibit less than 30% of the TCL, central isthmuses range from 30 to 50%, and entrance sites range from 50 to 70% [11]. Targeting these sites for ablation significantly enhances the probability of terminating the VT. Conversely, inner loops and adjacent bystander sites may demonstrate concealed fusion but do not directly sustain the arrhythmia [12]. Outer loop sites, while adjacent to the protective circuit and not anatomically constrained, and represent less ideal ablation targets [13].

The limitations of entrainment mapping include the need for hemodynamic stability during VT, the potential for pacing to alter the VT circuit, and challenges in identifying critical components in three-dimensional, nonischemic substrates [14].

#### Pace mapping

Pace mapping is particularly beneficial when activation mapping is constrained either by the difficulty of initiating focal arrhythmias or by the hemodynamic instability of re-entrant tachycardias. This technique entails pacing from various cardiac sites and comparing the morphology of the resultant QRS complex with that observed during the clinical VT [15]. A close resemblance between the paced and clinical QRS indicates proximity to the VT exit site, suggesting that the pacing site may be near the arrhythmia's origin. For focal arrhythmias, a site that generates an identical QRS complex is likely the origin point.

Advanced systems such as PASO (CARTO, Biosense Webster) and Automap Score Threshold (Precision, EnSite, Abbott) are employed to enhance pace mapping by quantitatively evaluating the similarity between the paced and clinical QRS, thereby improving the accuracy of the localization [16]. However, pace mapping's effectiveness is diminished in re-entrant circuits if pacing does not occur near the exit sites, resulting in significantly different paced QRS configurations [17]. Its spatial resolution is somewhat limited; accurate pace maps can appear even up to 26 mm from the actual origin [18]. The need for precise matching between the paced and clinical QRS, the subjective nature of the analysis, and the difficulty in accessing intramural circuits pose challenges to this method. Moreover, changes in QRS morphology during pacing may not definitively pinpoint the isthmus and could instead indicate remote bystander regions. Furthermore, during the ablation of VPDs or VTs originating from the left ventricular outflow tract, identifying the precise site of origin using conventional pace mapping can sometimes be exceptionally difficult. In such cases, premature extra-stimulus pace-mapping has emerged as a valuable adjunctive technique. By delivering premature stimuli, electrophysiologists can more accurately replicate the clinical VPD morphology and effectively identify the origin of the arrhythmia when standard pace mapping falls short [19].

Adjusting the pacing rate and amplitude is crucial as these factors can influence the QRS morphology, and typical bipolar stimulation may not accurately localize critical isthmuses [20]. Despite these limitations, pace mapping remains a valuable tool in the mapping arsenal, assisting in catheter positioning by enabling comparison of paced and clinical QRS configurations.

#### Substrate mapping

**Fig. 2:**
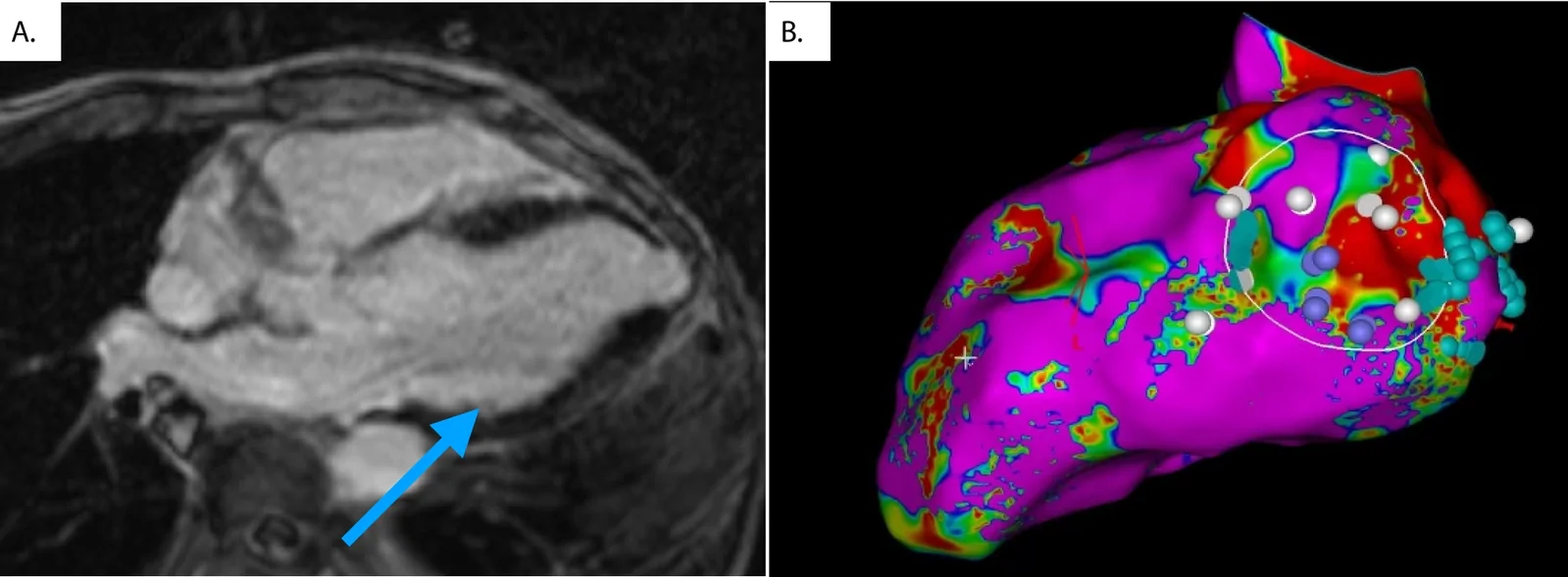
A. MRI showing lateral gadolinium enhancement, which correlated **B**. Endocardial low voltage and site of successful VT ablation

Substrate mapping is indispensable when VT cannot be assessed directly due to hemodynamic instability. This technique focuses on identifying abnormal electrograms during sinus or paced rhythms that signify scar-related arrhythmic substrates. Zones displaying low voltage, defined as less than 0.5 mV, typically indicate dense scar tissue, whereas areas showing values above 1.5 mV are considered normal [21]. This crucial data aids in precisely mapping and targeting the zones of dispersed electrical activity within scar borders, which are prone to reentrant VT mechanisms [22] (Fig. 2).

The integration of substrate mapping with traditional activation and entrainment mapping techniques significantly enhances the likelihood of effectively eliminating arrhythmic substrates and preventing future VT episodes. Substrate mapping employs EAM systems to delineate these critical areas for precise interventions effectively. This approach improves the immediate outcomes of ablation procedures by targeting the fibrotic tissues that facilitate reentry and helps reduce long-term arrhythmia recurrence and mortality [23].

Moreover, the utilization of high-density mapping tools has markedly advanced the precision of identifying and ablating local abnormal ventricular activities (LAVAs) and late potentials (LPs) [24]. These delayed activations serve as crucial indicators of slow-conduction zones within the myocardium and are essential targets in substrate-based ablation strategies [25]. Techniques such as Isochronal Late Activation Mapping (ILAM) are particularly valuable, as they provide insights into the precise locations of deceleration zones, aiding in the identification of ablation targets [26]. The continuous refinement of substrate mapping techniques has significantly enhanced the safety and efficacy of VT ablation, allowing for more tailored and effective treatment strategies in patients with complex cardiomyopathies [27].

#### Prominent mapping techniques using unipolar potentials

Unipolar voltage mapping provides a broader electrophysiological insight than bipolar mapping, and it has advanced significantly to include functional analyses [28]. These enhancements enable assessments of not only conduction abnormalities but also repolarization heterogeneity, which are critical elements in the formation of reentrant circuits [29].

A significant innovation in this domain is the reentry vulnerability index, which integrates activation time, activation-recovery interval (ARI), and repolarization time through unipolar potentials. This technique, developed by Orini M. and colleagues, achieves an accuracy rate of 72% for localizing VT origins, equating the effectiveness of deceleration zones identified by ILAM [26, 30]. Another notable advancement by Trayanova N. and his team showed that their method not only reduces the number of ablation sites and the size of ablation areas but also maintains effective outcomes [31]. This underscores the importance of precision in unipolar potential analysis, particularly in settings involving high-pass filter adjustments. Typical mapping systems use a 2 Hz high-pass filter to minimize baseline drift; however, this setting can alter the ARI, which is crucial for evaluating the ST-T segment. Studies that compare these standard settings with a lower 0.05 Hz setting (similar to those used in surface ECGs) have revealed significant differences in ARI measurements [32].

While ILAM predominantly captures near-field activity and may struggle with detecting intramural VT circuits, unipolar ARI mapping offers a wider scope, potentially identifying intramural VT isthmuses more effectively. This capability makes unipolar mapping a valuable tool in the complex landscape of VT ablation, providing essential data for targeting deep intramural pathways that are often elusive with other mapping techniques.

### The role of multimodality cardiac imaging techniques

Three-dimensional EAM has revolutionized the approach to VT ablation by targeting arrhythmogenic substrates. However, this technique often falls short due to its primary focus on endocardial and, occasionally, epicardial surfaces, which does not fully account for the complex three-dimensional nature of VT substrates, including variations in myocardial wall thickness, anatomical features, and scar depth. Furthermore, the effectiveness of EAM is compromised by inconsistent catheter contact during cardiac and respiratory motion, and by substrate locations beyond accessible endocardial surfaces [33].

To address these limitations, the integration of advanced imaging modalities with electrogram mapping has become indispensable. Intracardiac echography (ICE), CMR, and MDCT play pivotal roles in this integrated approach. These techniques provide high-resolution images crucial for the detailed delineation and characterization of scars, thereby enhancing the accuracy of identifying scar depth and distribution. Segmenting these detailed imaging findings and integrating them with EAM systems significantly improves the precision of mapping. This comprehensive approach not only reveals the extent of the scar but also the surrounding viable conducting tissues and border zones [33].

## Intracardiac echography in catheter ablation procedures

ICE has become an indispensable tool for guiding catheter ablation, offering real-time, detailed imaging of cardiac structures through a catheter-mounted echo transducer. Commonly used phased-array ICE probes, operating within a frequency range of 5–10 MHz, allow for deep penetration and precise imaging up to 16 cm, which is particularly suitable for comprehensive assessments from right-sided catheter positions, thus enhancing tissue visibility and resolution near the catheter tip [34].

**Fig. 3:**
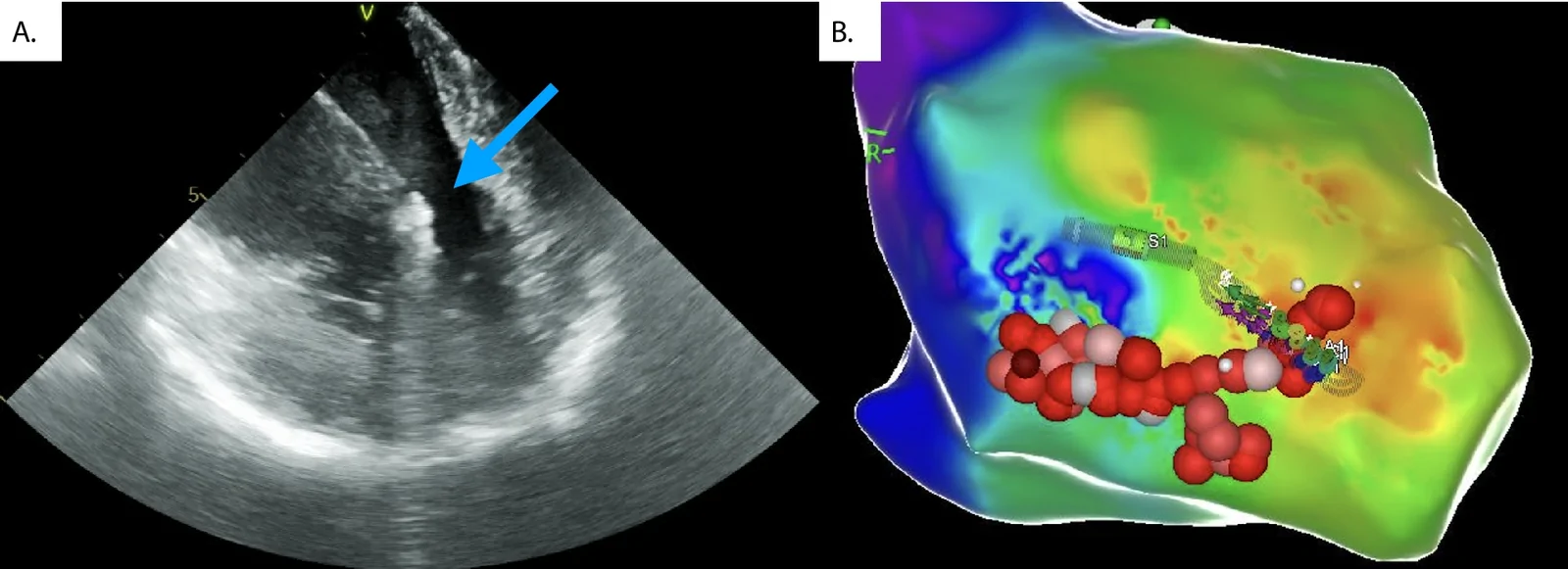
A. Intracardiac echocardiography shows RF catheter on head of papillary muscle which correlated to **B**. Successful ablation site of papillary PVC mediated VT

ICE is invaluable in optimizing catheter-tissue contact. It assists in navigating transseptal access and mapping potential VT substrates, which appear as echogenic regions often indicative of scar tissue. Additionally, ICE helps identify key anatomical landmarks, such as coronary artery ostia and valve annuli (Fig. 3). Its utility is further extended to real-time geometric mapping of challenging intracardiac areas, which proves particularly useful in ablating arrhythmias originating from the papillary muscles or para-Hisian regions [35]. Clinical outcomes from ICE-guided VT ablations demonstrate reduced complications such as cardiac tamponade and lower rates of hospital readmissions and repeat ablations within the first-year post-procedure [36].

Integrating ICE with 3D electro-anatomical mapping systems, such as CARTOSOUND®, significantly improves the efficiency of VT ablations [37]. This integration facilitates rapid anatomical reconstructions and detailed visualizations of cardiac outflow tracts and ventricular surfaces, which are crucial for effectively identifying and effectively targeting structural anomalies and aneurysms.

Moreover, ICE plays a critical role in the early detection of procedural complications, such as intracardiac thrombus formation and pericardial effusions [38]. It also enhances procedural safety by immediately detecting complications such as steam pops, indicated by sudden whitening of tissue, allowing for timely adjustments in RF energy application. This feature is particularly beneficial when ablation parameters like Ablation Index or Lesion Size Index are insufficient for assessing lesion effectiveness in the ventricles [37].

## Cardiac magnetic resonance in ventricular tachycardia ablation planning

CMR utilizes powerful magnetic fields and radiofrequency pulses to provide high-resolution images, making it a crucial tool for assessing cardiac structure and function. Particularly significant is the use of late gadolinium enhancement (LGE) for precisely identifying myocardial fibrosis [39]. Gadolinium contrast agents, which highlight areas of expanded extracellular space indicative of scar tissue or edema, are beneficial when imaged 10 to 15 min post-injection. This capability aids in differentiating cardiomyopathy etiologies and defining arrhythmogenic substrates before VT ablation.

Beyond LGE, CMR also employs T1, T2, and extracellular volume mapping to provide detailed characterization of structural abnormalities. This is invaluable in conditions like non-ischemic heart diseases [40], where traditional bipolar mapping may not adequately characterize arrhythmogenic sources deep within the myocardium, such as those in the interventricular basal septum. Research has shown that areas of transmural LGE correlate strongly with low voltage zones during EAM, confirming the linkage between dense scar tissues and electrical inactivity [41].

The role of CMR extends beyond diagnostic imaging to actively guide the VT ablation process. By enabling precise mapping of the myocardial scar’s range and distribution, CMR significantly enhances the planning and execution of ablation procedures. It is instrumental in determining the necessity of an epicardial approach, as delayed subepicardial enhancement has been shown to predict epicardial origins of VT accurately [42]. Moreover, CMR aids in accurately defining the border zone and heterogeneous tissue channels, differentiating between various intensities of LGE signals [43]. This capability is crucial for identifying potential channels within the scar that may serve as pathways for arrhythmia reentry. The 2019 European Heart Rhythm Association expert consensus underscores the importance of pre-procedural CMR, recommending its use to reduce recurrences in VT ablation procedures (Class II, level of evidence A) [1].

Despite its advantages, the effectiveness of CMR can be compromised by the presence of cardiac implantable electronic devices (CIEDs), which may introduce imaging artifacts. However, applying wideband LGE sequence acquisition techniques has proven effective in reducing these disturbances, ensuring the reliability of imaging results for pre-procedural planning [44–46].

## Multi-detector cardiac computed tomography in VT ablation planning

MDCT offers a robust alternative for assessing VT substrates when CMR is not feasible. Renowned for its high-resolution anatomical detail, MDCT excels at depicting myocardial wall thinning, myocardial scars, and border zones, all vital targets during VT ablations [47, 48].

MDCT provides comprehensive images of coronary arterial and venous anatomy, phrenic nerves, and epicardial fat distribution [49]. This is crucial, as epicardial fat can obstruct effective radiofrequency (RF) ablation, presenting unique challenges in procedural planning [50]. The ability of MDCT to quantify epicardial fat thickness and identify calcium deposits adds to its utility, especially in patients with ischemic heart disease, where thicker segments within myocardial scars, often below 5 mm in wall thickness, signify typical scarring that can harbor critical VT targets [51]. The high spatial resolution of MDCT surpasses that of LGE-CMR, enabling precise delineation of cardiac structures necessary for planning incredibly complex epicardial ablations. This method also explores new frontiers by developing late-iodine-enhanced MDCT to delineate fibrotic scar areas, correlating with successful ablation sites identified during EAM [51].

However, MDCT is not without drawbacks. The technique requires considerable radiation exposure and may encounter challenges such as imaging artifacts from CIEDs or distortions due to high heart rates or VPDs [52]. Advances like Photon-counting Computed Tomography are addressing these issues, improving spatial and contrast resolution, reducing image noise, and lessening radiation exposure [53]. This evolution in CT technology also introduces multi-parametric imaging capabilities, which could revolutionize cardiac imaging by enhancing tissue characterization and enabling more precise quantitative assessments.

## Enhancing VT ablation with segmentation software

Segmentation software, notably inHeart™ and ADAS 3D™, plays a role in integrating detailed cardiac imaging data into 3-D EAM systems. Utilizing CT or CMR scans, these tools enable the meticulous visualization of myocardial structures, coronary arteries, venous systems, and even CIED leads. They are proficient at generating scar maps, delineating arrhythmogenic isthmuses within fibrotic tissue, and significantly enhancing procedural planning [54].

The use of such software not only reduces the need for fluoroscopy, thereby decreasing radiation exposure, but also improves overall procedural outcomes. These segmentation tools' accuracy heavily depends on the quality of the initial imaging and their capability to align these images with the patient’s real-time cardiac anatomy [54]. However, time delays between the acquisition of images and their use in procedures can lead to discrepancies that may affect anatomical accuracy, impacting the procedure's success.

## Challenges and innovations in cardiac imaging for VT ablation

CMR and CT face several challenges in VT ablation procedures. Artifacts from implanted devices can significantly reduce the clarity of images, obscuring critical details of the affected ventricular segments. Furthermore, the efficacy of these imaging modalities heavily depends on local expertise's availability to ensure high-quality images, accurate interpretations, and meticulous integration with EAM systems. Additionally, the substantial costs associated with acquiring, implementing, and maintaining these advanced imaging tools and training professionals present considerable challenges [55].

Looking ahead, the future of cardiac imaging in VT ablation appears promising, with anticipated advancements in real-time imaging, epicardial space endoscopy, and enhanced integration software for EAM systems. Innovations such as 3-D and 4-D ICE and new mapping techniques like Voltage Excursion Duration Mapping (VEDUM), which highlights slow conduction areas, are poised to refine procedural accuracy [55]. The integration of artificial intelligence could also revolutionize this field by automating the localization of VT exit sites, potentially transforming the management and treatment of ventricular arrhythmias.

### Advancements in ablation technologies

The persistent challenge of VT recurrences in both ischemic and non-ischemic heart conditions is often attributed to the complex three-dimensional nature of arrhythmic circuits. These circuits include subendocardial, subepicardial, intramural, and transmural components. Traditional RF ablation, which generally impacts only up to 4 mm from the electrode, may not adequately achieve transmural scarring, leaving arrhythmic pathways intact [56]. New ablation technologies have been introduced to enhance efficacy, including bipolar ablation, ultra-low temperature cryoablation, needle-tipped electrode ablation, and PFA. These methods aim to create more extensive, penetrating lesions to improve ablation success rates.

Bipolar ablation leverages dual catheters connected to a radiofrequency generator on opposite myocardial surfaces (typically across the ventricular septum), facilitating comprehensive transmural lesions [57]. Advancements like decreased osmolarity irrigation and high-power energy delivery also create larger and deeper lesions, enhancing the scope and success of ablation procedures [58].

Ultra-low-temperature cryoablation is gaining attention for its efficacy in areas with thicker myocardial walls, such as the left ventricle, where walls often exceed 6 mm, challenging traditional RF ablation's effectiveness. Utilizing nitrogen at − 196 °C, this technique has shown promising results in early trials. However, these are limited by small sample sizes, highly selective patient criteria (only monomorphic VTs), and short follow-up periods [59]. It's exceptionally proficient for targeting papillary muscles and moderator bands, creating deeper and broader lesions than conventional RF methods can achieve [60, 61].

For particularly challenging cases, such as those involving intramyocardial substrates or VTs originating at summit locations, 98% ethanol injection through cardiac veins or coronary arteries offers a safe and effective bailout option, with demonstrated safety and excellent outcomes [62, 63]. Needle ablation has evolved with new catheter designs featuring retractable 27-gauge needles that allow for precise targeting and ablation of intramyocardial substrates, maintaining high success rates with minimal procedural risks [64–66].

Moreover, stereotactic ablative radiotherapy represents a cutting-edge, noninvasive alternative. Using a three-dimensional coordinate system based on CT, this method uses ionizing radiation to damage and kill cells within targeted heart zones. It offers a significant advance by treating VTs noninvasively, reducing the risks associated with traditional invasive procedures.

## Pulse field ablation: a paradigm shift in ventricular tachycardia management?

PFA is emerging as a transformative technology in cardiac electrophysiology, designed to manage VTs with a novel approach. PFA employs pulsed electric fields to induce irreversible electroporation, creating nanoscale disruptions in the cell membranes of cardiomyocytes [67]. Unlike traditional RF ablation, which relies on thermal damage, PFA does not, thereby minimizing the risk of collateral damage to adjacent tissues such as blood vessels, nerves, and the extracellular matrix. This approach enhances procedural safety and preserves cardiac structure [68, 69].

PFA's precise targeting capability allows it to produce extensive and deep lesions, addressing the complex arrhythmic circuits characteristic of both ischemic and non-ischemic cardiomyopathies. These circuits often involve multiple cardiac layers, including subendocardial, subepicardial, intramural, and transmural components. The depth and breadth of lesions created by PFA are crucial for effectively disrupting these complex pathways and reducing the incidence of VT recurrence [70, 71].

Despite its significant potential, applying PFA in VT ablation is still exploratory. Early clinical studies and trials have shown promising results regarding efficacy and safety, suggesting it could offer substantial improvements over existing ablation techniques. However, these studies also highlight the need for ongoing research to optimize the technology, understand its full capabilities, and establish standardized protocols for its use in VT ablation [72, 73].

While PFA has been studied extensively for atrial fibrillation, translating these findings to ventricular settings involves addressing different anatomical and pathological challenges. Adjustments in dosing, energy delivery, and understanding tissue responses in the ventricular myocardium are critical areas of ongoing research [73, 74]. Experimental studies have demonstrated lesion depths in the ventricles ranging from 4 to 8 mm with PFA, suggesting that this technique may be comparable to, or even more profound than, RF ablation [75]. Previous case series have demonstrated a procedural success rate of over 80% with no recurrence of VTs [76]. Notably, contact force, which was once deemed of limited importance in the early days of PFA, is now recognized as essential for creating deep and large lesions with this technique [77].

Improvements in catheter design are being evaluated to increase maneuverability and enhance ablation accuracy [78]. The future of PFA in VT ablation looks promising, with potential expansions in its application as safety and efficacy are further established through clinical trials. The introduction of PFA is poised to significantly impact the management of complex ventricular arrhythmias, potentially becoming a standard care component, provided further validation supports its benefits [79, 80].

## Surgical management of ventricular tachycardias

Surgical management of VT is the most invasive yet often the most effective approach, typically reserved for cases refractory to medical therapy, catheter ablation, and ICD therapy. Surgical intervention is less commonly utilized due to advancements in catheter ablation but remains vital for treating structural heart diseases like ischemic cardiomyopathy or arrhythmogenic right ventricular cardiomyopathy [81].

Surgical intervention is indicated in several scenarios, including persistent VT despite AAD and catheter ablation, post-myocardial infarction (MI) scar-related VT inaccessible by catheter methods, and in patients with complications such as left ventricular aneurysm or hypertrophic cardiomyopathy. It is particularly pertinent for patients who are unable to tolerate ICD shocks or where other therapies have proved ineffective or inaccessible.

The surgical approach typically involves the subxiphoid and limited thoracotomy techniques, providing access to various ventricular segments and often assisted by video thoracoscopy. This access is crucial for carrying out procedures such as endocardial and epicardial resection, especially in cases where VT originates from scarred myocardium [81]. Surgical approaches are particularly applicable to other cardiac surgeries, such as left ventricular assist device implantation, which complicates subsequent epicardial access [82]. Additionally, aneurysmectomy is performed for VT associated with left ventricular aneurysm, usually post-MI, where resection of the aneurysm along with scar modification helps eliminate the arrhythmogenic substrate. This is often coupled with cryotherapy or RF ablation to eradicate any residual arrhythmogenic tissue. Intraoperative cryotherapy is mainly utilized to target VT circuits in non-resectable myocardial scars, often in conjunction with coronary artery bypass grafting (CABG) for patients with ischemic heart disease.

Hybrid surgical ablation techniques have also been explored for patients with previous cardiac surgeries or unsuccessful percutaneous access [83]. Despite potential complications like pain, bleeding, and infection, these approaches have shown efficacy comparable to percutaneous methods [84]. The work of Kunkel et al. has been particularly notable, focusing on patients with refractory VT where previous surgical or pericardial interventions complicated epicardial access, demonstrating significant reductions in VT episodes from a median of 15 to 0 in the month post-procedure [85].

While the procedural risk associated with surgical VT treatment is higher than that of catheter ablation, it provides definitive arrhythmia control for select patients, especially those undergoing concurrent cardiac surgeries such as CABG or aneurysmectomy. Postoperative ICD implantation is typically recommended due to the risk of recurrent VT.

## Ventricular tachycardia ablation in patients with left ventricular assist devices

The growing population of patients supported by Left Ventricular Assist Devices (LVADs) presents a unique set of challenges for VT management. Recent clinical consensus statements from the European Heart Rhythm Association and the Heart Failure Association of the ESC formally recommend catheter ablation for LVAD recipients experiencing symptomatic, recurrent ventricular arrhythmias that are refractory to antiarrhythmic drugs or ICD reprogramming [86]. Early pooled analyses of single-center experiences established the foundational safety and feasibility of VT ablation in LVAD patients [87]. Extensive multicenter experience has further detailed the complex nature of VT in continuous-flow LVAD recipients, demonstrating that high rates of acute procedural success can be achieved when targeting the underlying arrhythmogenic substrate [88].

A contemporary 2025 meta-analysis comprising over 300 LVAD recipients demonstrated that catheter ablation is a feasible and safe intervention, showing a low overall procedural complication rate of 7.4% [89]. Despite the high clinical complexity of this cohort, acute non-inducibility is achieved in over 61% of cases, though long-term VT recurrence remains a challenge at 38% [89]. The arrhythmogenic substrate in LVAD recipients is primarily driven by scar-related macro-reentrant circuits (80%), with inflow cannula-related reentry accounting for approximately 18% of cases; these critical scars are most frequently localized to the apex and peri-cannular region (30%), the septum (30%), and the anterior wall (27%) [89].

The improved safety profile of contemporary LVADs during VT ablation is particularly significant when contrasted with older continuous-flow generations, which were historically associated with an increased risk of pump thrombosis and cardioembolic events following ablation procedures [90]. The advent of the fully magnetically levitated HeartMate 3 (HM3) device has significantly improved the safety profile of these procedures. VT ablation in patients supported by the HM3 system has demonstrated a highly favorable safety profile, with studies highlighting a notable absence of post-ablation device thrombosis or thromboembolic events [89, 91]. However, the HM3 introduces significant procedural challenges during VT ablation, most notably severe electromagnetic interference that can obscure catheter visualization and disrupt standard 3D electro-anatomical mapping in over half of cases [89, 92]. To overcome this, electrophysiologists increasingly rely on complementary imaging, such as intracardiac echocardiography, and the strategic placement of mapping patches to safely navigate the inflow cannula and accurately define the arrhythmic substrate [92].

## Conclusion

The management of VTs continues to evolve rapidly, driven by significant technological leaps in mapping, multimodality imaging, and novel energy sources like pulse field ablation. By shifting from purely anatomical to highly detailed, functional, and substrate-based approaches, electrophysiologists can now target complex intramural and epicardial circuits with unprecedented precision.

Although these innovations were developed primarily for VT, their application to ventricular fibrillation (VF) is a highly promising frontier. Current high-resolution substrate mapping and non-invasive imaging techniques can already identify the localized structural anomalies, tissue channels, and Purkinje-related triggers responsible for initiating and sustaining VF. Ultimately, as novel tools like VEDUM and PFA mature, their ability to precisely map and modify these complex VF substrates will pave the way for more definitive arrhythmia management.

## Data Availability

No datasets were generated or analysed during the current study.

## Abbreviations

CIED: Cardiac Implantable Electronic Devices
CMR: Cardiac Magnetic Resonance
EAM: 3-Dimensional Electro-anatomical Mapping
ICD: Implantable Cardioverter-Defibrillators
ICE: Intracardiac Echocardiography
LVAD: Left Ventricular Assist Device
MDCT: Multidetector Cardiac Computed Tomography
PFA: Pulse Field Ablation
RF: Radiofrequency
VF: Ventricular Fibrillation
VPD: Ventricular Premature Depolarizations
VT: Ventricular Tachycardia
